# Integrative study reveals the prognostic and immunotherapeutic value of CD274 and PDCD1LG2 in pan-cancer

**DOI:** 10.3389/fgene.2022.990301

**Published:** 2022-10-06

**Authors:** Xuan Zhou, Yu Wang, Jianwei Zheng, Sinan Wang, Chao Liu, Xiaofeng Yao, Yu Ren, Xudong Wang

**Affiliations:** ^1^ Department of Maxillofacial and Otorhinolaryngological Oncology, Tianjin Medical University Cancer Institute and Hospital, Key Laboratory of Cancer Prevention and Therapy, Tianjin Cancer Institute, National Clinical Research Center of Cancer, Tianjin, China; ^2^ Department of Gastroenterology and Hepatology, Tianjin Medical University General Hospital, Tianjin Gastroenterology and Hepatology Institute, Tianjin Medical University, Tianjin, China; ^3^ Department of Genetics, School of Basic Medical Sciences, Tianjin Medical University, Tianjin, China

**Keywords:** CD274, PDCD1LG2, pan-cancer, prognostic biomarker, cancer immunity

## Abstract

**Background:** Disorders of CD274 and PDCD1LG2 contribute to immune escape in human cancers, and treatment with anti-programmed death receptor 1 (PD-1) has been widely used in recurrent or metastatic tumors. However, integrated studies considering CD274 and PDCD1LG2 across cancers remain limited.

**Materials and Methods:** Differences in expression levels of CD274 and PDCD1LG2 were analyzed in diverse cancer types using The Cancer Genome Atlas (TCGA) and Genotype-Tissue Expression (GTEx) databases. The clinical information and matched expression profiles of TCGA patients were obtained to determine the prognostic value of CD274 and PDCD1LG2. Moreover, correlations between CD274 and PDCD1LG2 and the immune signature were analyzed by exploring the TIMER2 and TISIDB databases. We also investigated correlations between CD274 and PDCD1LG2 and immunotherapeutic biomarkers, including mismatch repair (MMR), tumor mutation burden (TMB), microsatellite instability (MSI), and DNA methylation.

**Results:** Expression levels of CD274 and PDCD1LG2 varied across multiple cancer types. CD274 and PDCD1LG2 not only impacted the prognosis of patients with cancer but were associated with clinical characteristics (lymph node metastasis, tumor stage, and sex) in kidney renal papillary cell carcinoma, thyroid carcinoma, and some other cancer types. Typically, CD274 and PDCD1LG2 could be strongly correlated with macrophages, dendritic cells, neutrophils, and CD8^+^ T-cells. Furthermore, CD274 and PDCD1LG2 expression were associated with various immunosuppressive biomarkers, such as CTLA4, TIGIT, and LAG3. In addition, CD274 and PDCD1LG2 were significantly associated with MMR, TMB, MSI, and DNA methylation. Finally, enrichment analysis confirmed that CD274 and PDCD1LG2 were associated with numerous biological pathways, such as: “Activation of Immune Reactions” and “Epithelial-Mesenchymal Transition,” suggesting that CD274 and PDCD1LG2 play crucial roles in cancer immunity and tumor metastasis.

**Conclusion:** CD274 and PDCD1LG2 play critical roles in cancer progression and immune response and could serve as effective biomarkers to predict the prognosis and immune signature of cancer.

## Introduction

Cancer is a lethal disease characterized by genetic mutations and epigenetic disorders (Porta-Pardo et al., 2017). Recently, genomic sequencing in large-scale studies, such as The Cancer Genome Atlas (TCGA) (Weinstein et al., 2013) and the International Cancer Genome Consortium (ICGC) (Bailey et al., 2018), has expanded our understanding of cancer genomics. A recent pan-cancer study has reported notable differences between the genomes, epigenomes, and transcriptomes in 33 types of cancer (Hoadley et al., 2018). For example, the PIK3CA mutant-driven receptor tyrosine kinase (RTK) signaling pathway is responsible for metastasis, proliferation, and drug resistance in multiple human cancers (Imperial et al., 2019; Tang et al., 2020). Thus, a pan-cancer analysis affords a new approach to identifying potential targets for cancer (Hu et al., 2019).

Comprehensive genomic studies assessing programmed death receptor 1 (PD-1) have confirmed that PD-1 is a crucial biomarker for immunity (Liu et al., 2020). CD274 (also called programmed death ligand 1 [PD-L1] and B7-H1) and PDCD1LG2 (also called programmed death ligand 2 [PD-L2] and B7-DC) are two ligands of PD-1 (Zou and Chen, 2008). Combining PD-L1 or PD-L2 with PD-1 triggers exhausted T cell formation and finally leads to cancer progression (Dammeijer et al., 2020). Moreover, high PD-L1 expression contributes to cancer drug resistance and recurrence (Benci et al., 2016). In addition, previous studies by our team have confirmed that patients with high PD-L2 levels in locally advanced head and neck squamous cell carcinoma (HNSC) have a worse prognosis, and PD-L2 expression levels are related to the expression of immune-related proteins (Qiao et al., 2021). The tumor proportion score (TPS) or combined positive score (CPS), defined by immunohistochemical (IHC) staining, was used to predict the anti-PD-1 therapeutic effect (de Ruiter et al., 2021). However, some patients with cancer presenting relatively low PD-L1 levels still exhibit a favorable response to anti-PD-1 therapy (Riaz et al., 2017). PD-L2 functions as an important target with therapeutic potential in HNSC (Solomon et al., 2018). Qiao et al. (2021) have suggested that immunoscore evaluation by the PD-L1/PD-L2 combination was more effective in predicting the immune signature.

Although a variety of previous studies have demonstrated the expression and functions of CD274 and PDCD1LG2 in certain cancer types, these studies tended to focus on individual cancer types, which restricted the co-analysis of CD274 and PDCD1LG2. Herein, we screened the co-expression level of CD274/PDCD1LG2 using TCGA and Genotype-Tissue Expression (GTEx) databases and found that these expression levels were related to treatment outcomes in patients with cancer. Moreover, the potential impact of CD274 and PDCD1LG2 on the immune microenvironment and its biological functions was further explored. Overall, these data indicated that CD274 and PDCD1LG2 could be established as biomarkers of therapeutic value in various cancers.

## Materials and methods

### Data collection

The RNA-seq and corresponding clinical profiles of 33 tumor types and normal tissues were downloaded from TCGA and GTEx databases. R software (version 4.1.0) (https://www.r-project.org/) was employed in this analysis, and the R package “ggplot2” was applied to draw box plots. The Wilcoxon test was conducted to detect differences in expression between the two sets.

### Diagnostic analysis and prognostic analysis

The receiver operating characteristic (ROC) curve was used to evaluate the pan-cancer diagnostic value of CD274 and PDCD1LG2. An area under the curve (AUC) value >0.7 was considered to indicate a certain accuracy. RNA-seq data and related clinical data were obtained from the XENA database (https://xena.ucsc.edu/) and then transformed into transcripts per million reads (TPM). Boxplots were generated by “ggplot2” to compare the expression differences between the two groups.

Next, to probe the prognostic value of CD274 and PDCD1LG2 in 33 cancer types, R software packages “survminer” and “survival” were used for univariate Cox regression (uniCox). The overall survival (OS), disease-specific survival (DSS), and progression-free interval (PFI) were analyzed. A 50% cut-off value was regarded as the threshold in the Kaplan–Meier (K–M) survival analysis. The R package “ggplot2” was used to generate forest plots.

### Immune infiltration evaluation

First, gene correlations with immune cells were explored *via* the TIMER2 website (TIMER2.0 (cistrome.org). The TIMER and CIBERSORT algorithms were used to estimate immune infiltration. After, we investigated the potential association between CD274/PDCD1LG2 levels and immunosuppressive molecules, major histocompatibility complex (MHC) molecules, and chemokines using the TISIDB web server (http://cis.hku.hk/TISIDB/index.php). We determined the lowest and highest correlations among the results.

Then, Estimation of Stromal and Immune cells in Malignant Tumor tissues using Expression data (ESTIMATE) was used to predict the proportion of infiltrating stromal/immune cells in tumor tissues (Yoshihara et al., 2013). The stromal score, immune score, and tumor purity were calculated.

The relationship between CD274/PDCD1LG2 and tumor mutation burden (TMB) or microsatellite instability (MSI) was analyzed. The “Maftools” R package was used to calculate the TMB. TMB combined with PD-L1 expression has been shown to be a useful biomarker for selection of immune checkpoint blockade (ICB) in certain cancer types (Chan et al., 2019). Moreover, MSI was observed in several cancers, most commonly colorectal cancer, endometrial cancer, and gastric adenocarcinoma (Bonneville et al., 2017). Thus, we focused on these three cancer types to discuss the relationship between CD274/PDCD1LG2 and the efficacy of ICB treatment. Besides, the correlations between CD274/PDCD1LG2 levels and TMB or MSI were visualized using radar diagrams.

Furthermore, we analyzed the correlation between CD274/PDCD1LG2 and mismatch repair (MMR) genes. The expression data for DNMT1, DNMT3A, DNMT3B, and DNMT3L were obtained to evaluate the relationship between the four methyltransferases and CD274/PDCD1LG2.

### Functional analysis

Gene Ontology (GO) and Kyoto Encyclopedia of Genes and Genomes (KEGG) analyses were performed to evaluate the functions of CD274 and PDCD1LG2. Gene set enrichment analysis (GSEA) was used to explore the biological pathways of high-expression CD274/PDCD1LG2 groups. “ClusterProfiler” was used for analysis (Yu et al., 2012), showing the first 10 items of GO and KEGG pathways as well as the first 4 items of GSEA results. Normalized *p* < 0.05 were defined as biologically significant.

### Statistical analysis

R software was used for data analysis. Comparisons between groups were performed using the Wilcoxon test. The hazard ratio and Cox *p*-value were used to compare OS, DSS, and PFI across cancers. Spearman’s or Pearson’s tests were used for the correlation analysis (*p < 0.05, **p < 0.01, ***p < 0.001).

## Results

### CD274 and PDCD1LG2 expression in pan-cancer

We summarized the analysis and exhibited in Supplementary Figure S1 for a more comprehensive prospect. The TCGA and GTEx databases declared variable CD274 and PDCD1LG2 expression levels across 33 cancer types. According to the comparison of expression differences, CD274 and PDCD1LG2 were found both upregulated in lymphoid neoplasm diffuse large B-cell lymphoma (DLBC), esophageal carcinoma (ESCA), glioblastoma multiforme (GBM), HNSC, kidney renal clear cell carcinoma (KIRC), acute myeloid leukemia (LAML), brain lower grade glioma (LGG), pancreatic adenocarcinoma (PAAD), stomach adenocarcinoma (STAD), testicular germ cell tumors (TGCT) and thymoma (THYM). On the contrary, both of them were downregulated in adrenocortical carcinoma (ACC), lung adenocarcinoma (LUAD), lung squamous cell carcinoma (LUSC), prostate adenocarcinoma (PRAD), uterine corpus endometrial carcinoma (UCEC), and uterine carcinosarcoma (UCS).

**FIGURE 1:**
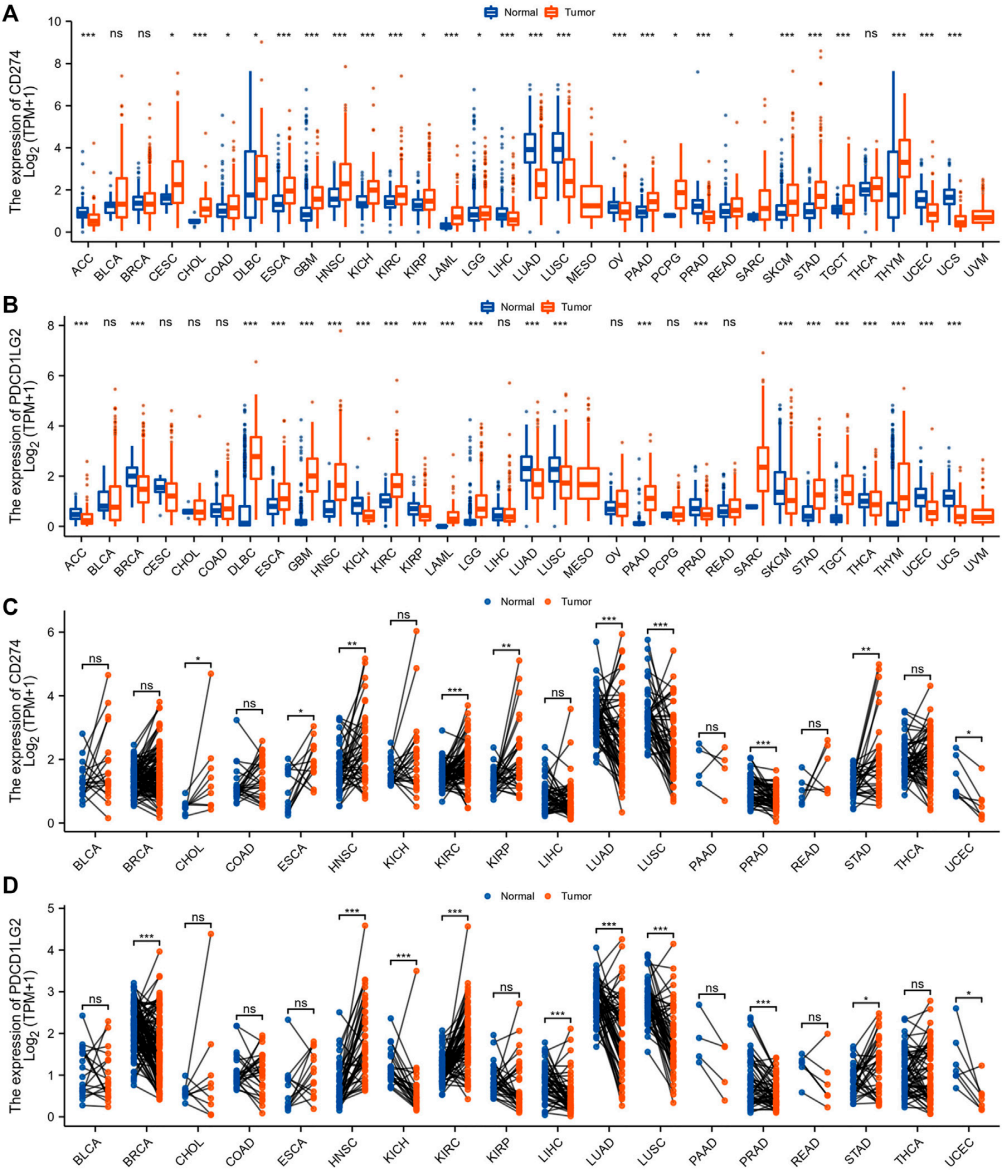
CD274 and PDCD1LG2 expression in different cancer types. **(A,B)** CD274 and PDCD1LG2 levels in TCGA tumor samples and normal tissues with samples in the GTEx database as controls. **(C,D)** Expression of CD274 and PDCD1LG2 in tumor samples and paired adjacent tissues from the TCGA. (*p < 0.05, **p < 0.01, ***p < 0.001).

CD274 was also upregulated in cervical squamous cell carcinoma (CESC), cholangiocarcinoma (CHOL), colon adenocarcinoma (COAD), kidney chromophobe (KICH), kidney renal papillary cell carcinoma (KIRP), pheochromocytoma and paraganglioma (PCPG), rectum adenocarcinoma (READ), and skin cutaneous melanoma (SKCM) and downregulated in liver hepatocellular carcinoma (LIHC) and ovarian serous cystadenocarcinoma (OV), whereas PDCD1LG2 level was decreased in breast cancer (BRCA), SKCM, and thyroid carcinoma (THCA) (Figures 1A,B). The CD274 and PDCD1LG2 levels in tumor tissues and paired adjacent normal tissues in the TCGA are displayed in Figures 1C,D.

### Diagnostic value of CD274 and PDCD1LG2

**FIGURE 2:**
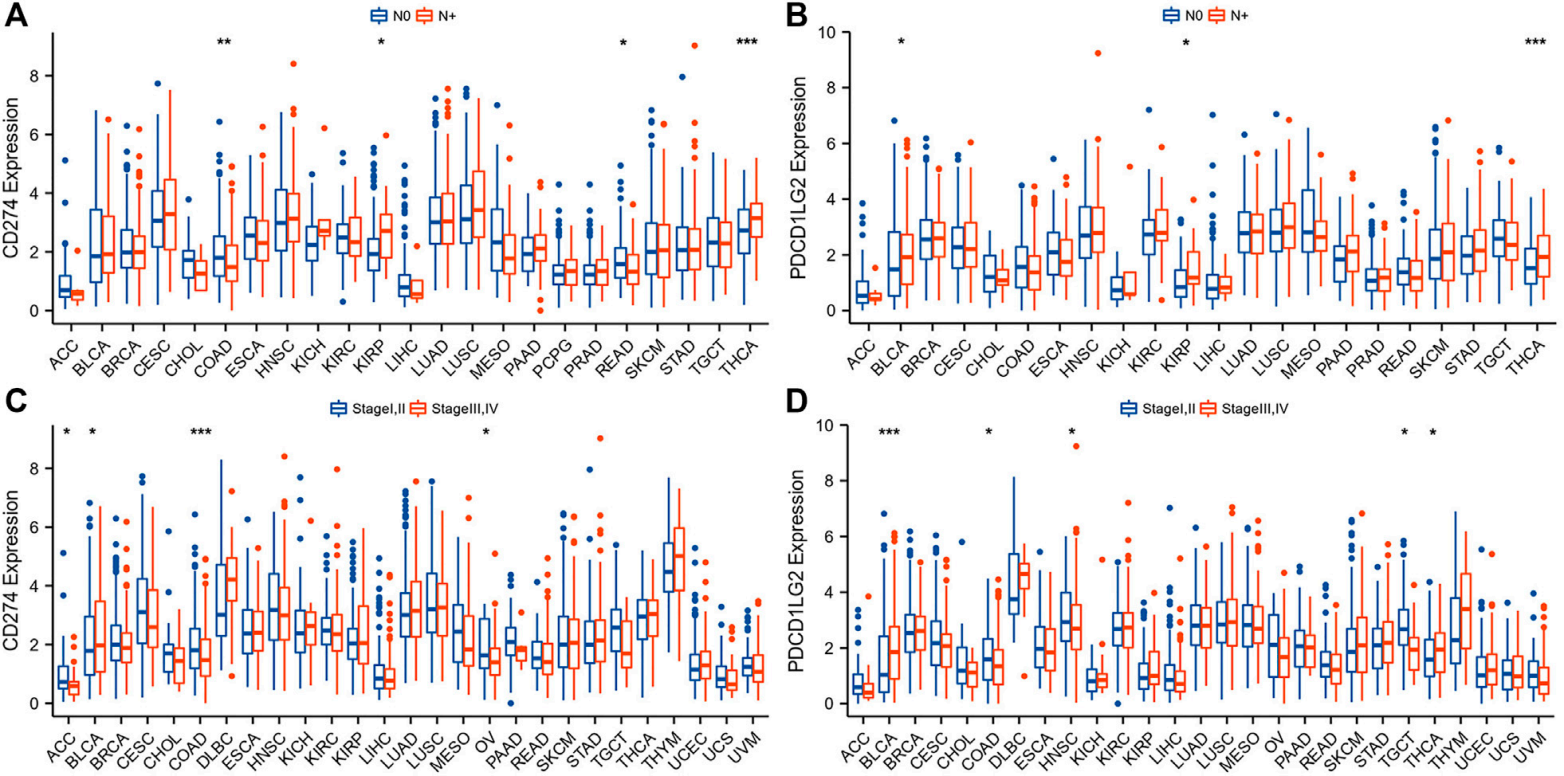
Association between CD274/PDCD1LG2 levels and clinical characteristics. **(A,B)** N stage. **(C,D)** Clinical stage. (“N+“: primary tumor with lymph node metastasis; *p < 0.05, **p < 0.01, ***p < 0.001).

To investigate the clinical value of these two immunological biomarkers, we compared CD274/PDCD1LG2 levels with N stage and clinical stage. The results indicated that high CD274 and PDCD1LG2 expression levels were associated with lymph node metastasis in KIRP and THCA (Figures 2A,B), and that CD274 and PDCD1LG2 levels were associated with the tumor stage of several cancers, including ACC, BRCA, bladder urothelial carcinoma (BLCA), COAD, HNSC, OV, SKCM, TGCT, and THCA (Figures 2C,D; Supplementary Figures S2A,B). In addition, CD274 and PDCD1LG2 expression has been observed to be sex-dependent in HNSC and some other cancer types (Supplementary Figures S2C,D).

The ROC curve was used to evaluate the diagnostic value of CD274 and PDCD1LG2 in pan-cancer analysis. For CD274, 14 cancer predictions were accurate (Supplementary Figure S3A), including those for ACC (AUC = 0.741), CHOL (AUC = 0.812), ESCA (AUC = 0.814), GBM (AUC = 0.785), HNSC (AUC = 0.720), LAML (AUC = 0.840), LUAD (AUC = 0.783), LUSC (AUC = 0.711), PAAD (AUC = 0.743), PRAD (AUC = 0.736), STAD (AUC = 0.748), THYM (AUC = 0.701), UCEC (AUC = 0.755), and UCS (AUC = 0.910). For PDCD1LG2, 19 cancer predictions were accurate (Supplementary Figure S3B), including those for ACC (AUC = 0.703), BRCA (AUC = 0.744), DLBC (AUC = 0.877), ESCA (AUC = 0.743), GBM (AUC = 0.985), HNSC (AUC = 0.849), LAML (AUC = 0.973), LGG (AUC = 0.887), LUAD (AUC = 0.836), LUSC (AUC = 0.801), KICH (AUC = 0.919), KIRC (AUC = 0.767), KIRP (AUC = 0.720), PAAD (AUC = 0.951), READ (AUC = 0723), TGCT (AUC = 0.913), THYM (AUC = 0.794), UCEC (AUC = 0.785), and UCS (AUC = 0.845).

### Prognostic value of CD274 and PDCD1LG2

**FIGURE 3:**
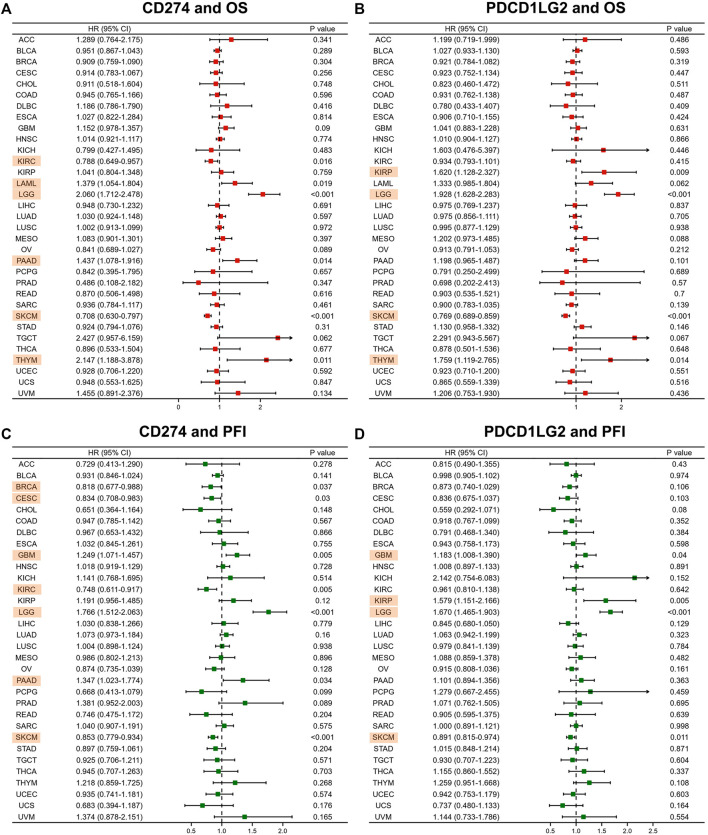
CD274/PDCD1LG2 is related to different prognoses. Forest plot showing the association of CD274 and PDCD1LG2 with **(A,B)** OS and **(C,D)** PFI. Cancers with *p* < 0.05 are highlighted.

Forest plots were generated after performing Cox regression analysis. CD274 was proved a prognostic biomarker for OS in KIRC (*p* = 0.016), LAML (*p* = 0.019), LGG (*p* < 0.001), PAAD (*p* = 0.014), SKCM (*p* < 0.001) and THYM (*p* = 0.011) (Figure 3A). Similarly, PDCD1LG2 was also a prognostic factor for OS in KIRP (*p* = 0.009), LGG (*p* < 0.001), SKCM (*p* < 0.001) and THYM (*p* = 0.014) (Figure 3B). Next, the uniCox analysis exhibited the results of DSS and PFI. The CD274 level was proved a prognostic biomarker for PFI in BRCA, CESC, GBM, KIRC, LGG, PAAD, and SKCM (Figure 3C). The PDCD1LG2 level was also a prognostic factor for PFI in GBM, KIRP, LGG and SKCM (Figure 3D). Supplementary Figures S4A,B showed the relationship between CD274/PDCD1LG2 and DSS.

**FIGURE 4:**
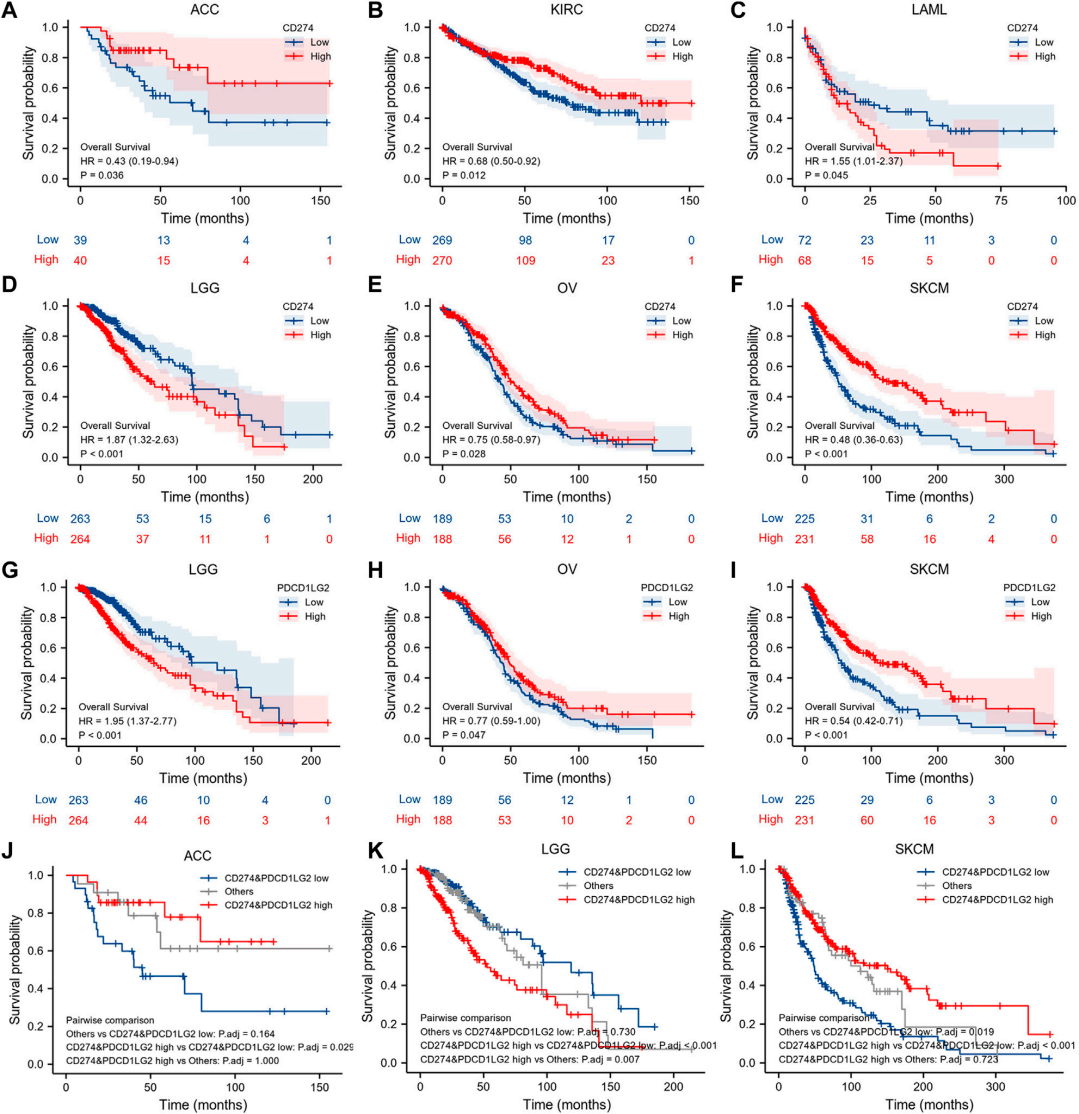
K–M survival analysis of CD274 and PDCD1LG2. **(A–F)** Relationship between CD274 level and OS. **(G–I)** Relationship between PDCD1LG2 level and OS. Co-expression survival analysis of CD274 and PDCD1LG2 in **(J)** ACC, **(K)** LGG, and **(L)** SKCM.

We also employed K–M survival analysis to identify the prognostic factors for CD274 and PDCD1LG2. In ACC (*p* = 0.036), KIRC (*p* = 0.012), OV (*p* = 0.028), and SKCM (*p* < 0.001), patients with high CD274 expression had longer OS than those with low CD274 expression (Figures 4A,B,E,F), but in LAML (*p* = 0.045) and LGG (*p* < 0.001), patients with high CD274 expression exhibited shorter OS (Figures 4C,D). Meanwhile, in OV (*p* = 0.047) and SKCM (*p* < 0.001), patients with high PDCD1LG2 levels exhibited longer OS than those with low PDCD1LG2 levels (Figure 4H,I); however, in LGG (*p* < 0.001), patients with high PDCD1LG2 expression had shorter OS (Figure 4G). Furthermore, we investigated the co-expression survival analysis of CD274 and PDCD1LG2 in pan-cancer and found that both low expression of CD274 and PDCD1LG2 had better prognosis in ACC (*p* < 0.019) (Figure 4J) and SKCM (*p* < 0.001) (Figure 4L), and both high expression of CD274 and PDCD1LG2 had worse prognosis in LGG (*p* < 0.001) (Figure 4K).

### Correlation between CD274/PDCD1LG2 and immune infiltration

**FIGURE 5:**
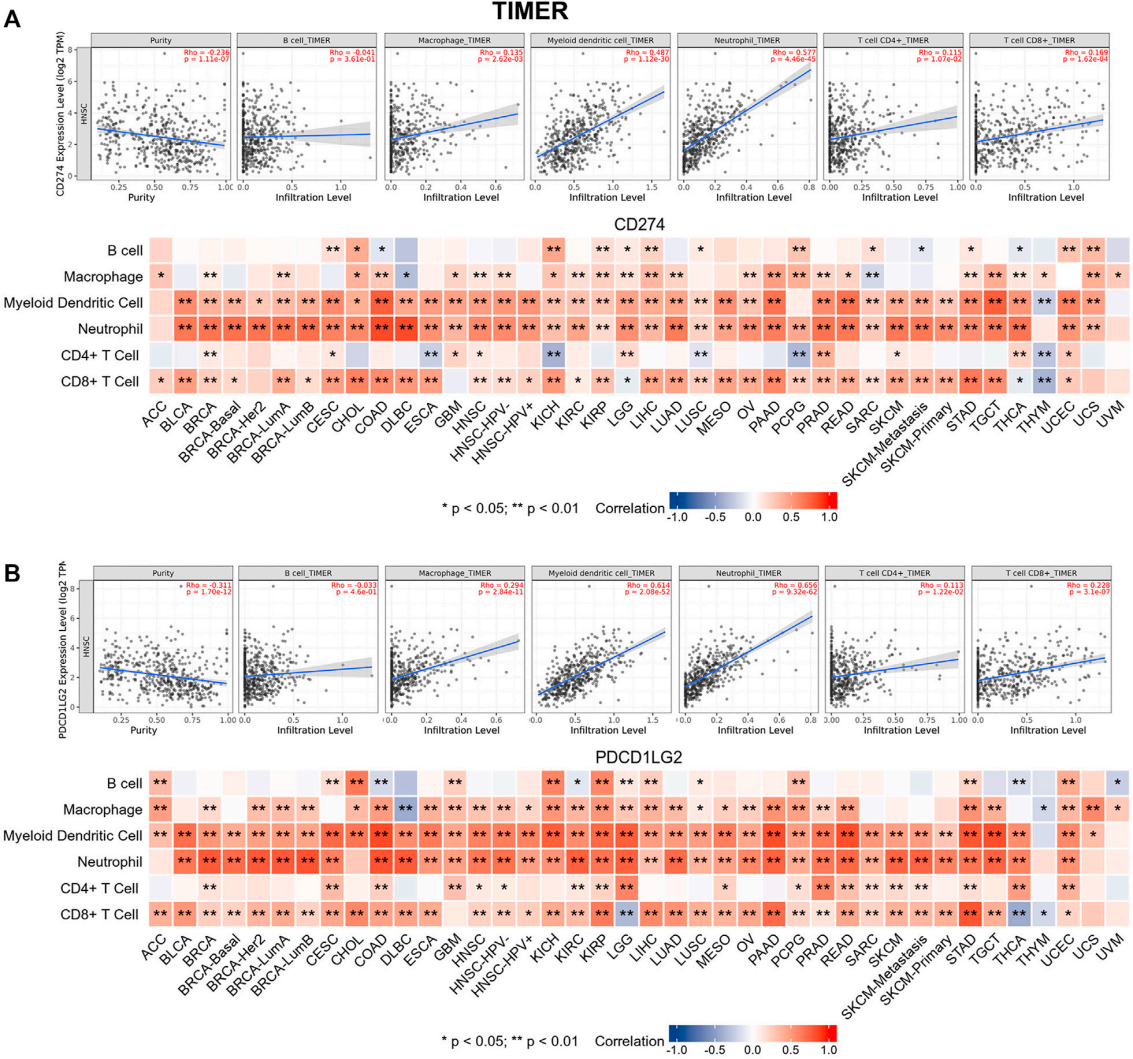
Relationships between **(A)** CD274 and **(B)** PDCD1LG2 expression and six types of immune cells across cancers determined using TIMER. Representative results in HNSC are shown at the top.

**FIGURE 6:**
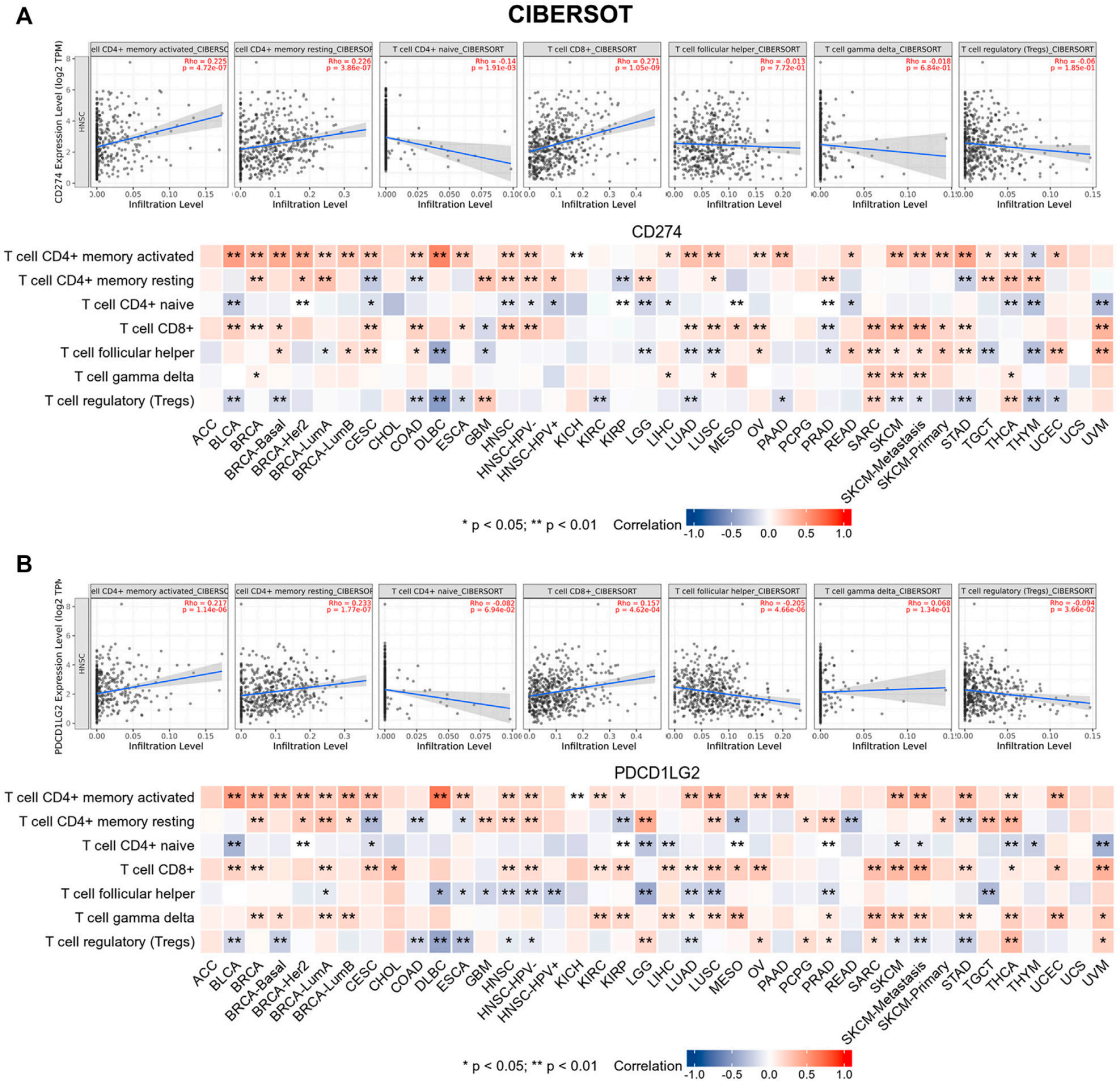
CIBERSORT predicted the relationship between **(A)** CD274 and **(B)** PDCD1LG2 expression and seven T-cell subtypes. Representative results in HNSC are shown at the top.

Tumor-infiltrating immune cells are generally and aberrantly expressed within the tumor microenvironment (TME), and were widely validated to promote immune escape and finally trigger tumor progression. Thus, the relationship between CD274/PDCD1LG2 and the degree of immune cell infiltration was explored, based on TIMER, the data showed that CD274/PDCD1LG2 levels were positively associated with macrophages, dendritic cells, neutrophils, and CD8^+^ T cells in most cancers (Supplementary Table S1). Notably, CD274 expression was negatively associated with CD4^+^ T cells in ESCA, KICH, LUSC, PCPG, and THYM, while CD274 and PDCD1LG2 levels were negatively correlated with CD8^+^ T cells in LGG, THCA, and THYM. The associations of six immune cells with the levels of CD274 and PDCD1LG2 are displayed in the bottom panel of Figures 5A,B, respectively. Since we found that the correlation between T cells and CD274 and PDCD1LG2 levels was diverse in generalized carcinoma, the relationship between CD274/PDCD1LG2 and 7 types of T cells was analyzed by the CIBERSORT algorithm (Supplementary Table S2). In most cancers, CD274 and PDCD1LG2 showed significantly positive relationship with memory-activated CD4^+^ T cells but a negative correlation with naive CD4^+^ T cells (Figure 6A,B).

**FIGURE 7:**
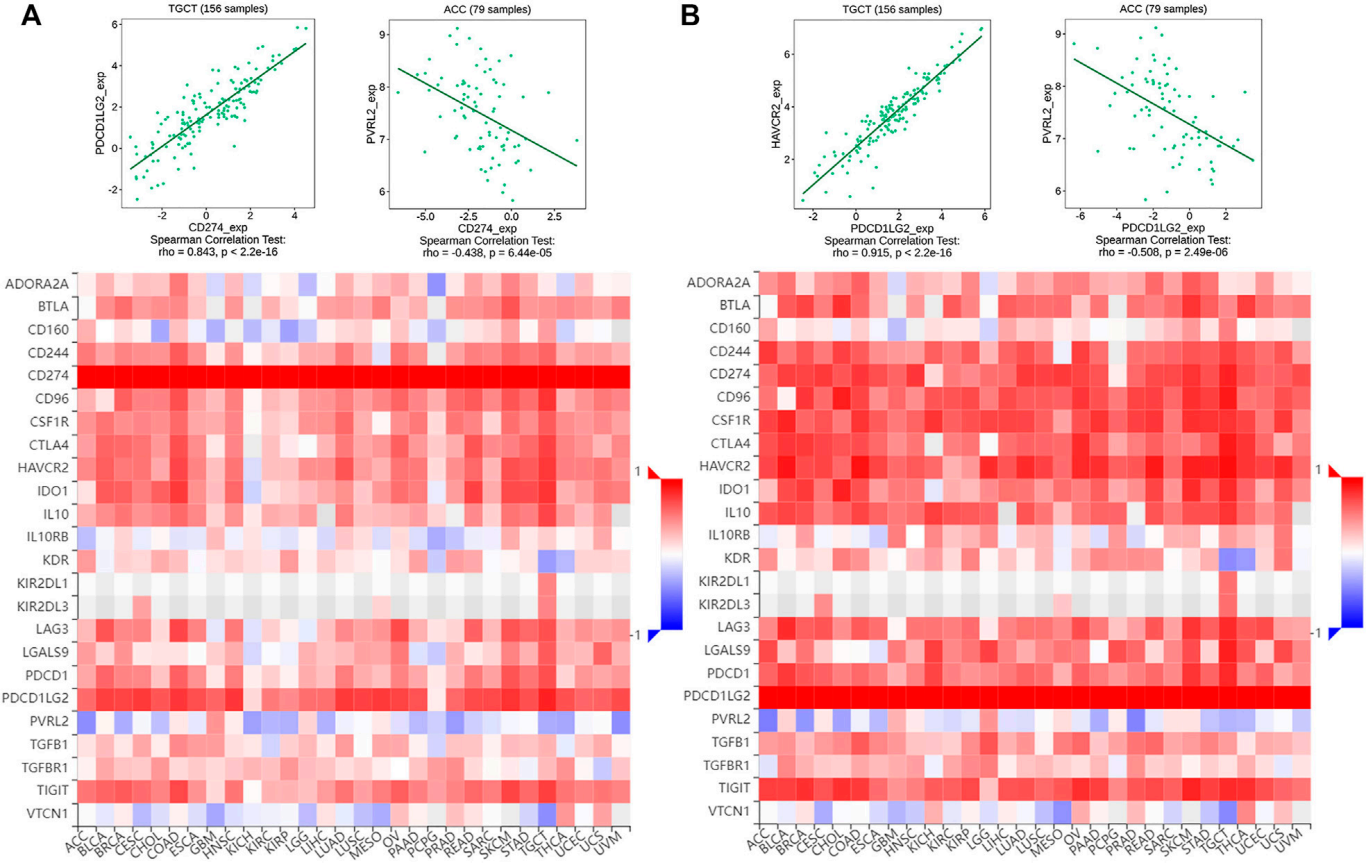
Correlation of multiple immunosuppressive genes including CD274/PDCD1LG2. Heatmap of the correlation between **(A)** CD274 and **(B)** PDCD1LG2 and immunosuppressive genes. Red represents positive correlations, and blue represents negative correlations. The strongest correlation is shown at the top.

Owing to their immunosuppressive function, CD274 and PDCD1LG2 levels were found to be positively correlated with most immunosuppressive molecules across cancers, and both had a significant correlation with TGCT. However, CD274 and PDCD1LG2 were negatively correlated with PVRL2 and not significantly correlated with KIR2DL1 or KIR2DL3 (Figures 7A,B). Furthermore, CD274/PDCD1LG2 levels were strongly associated with MHC molecules (Supplementary Figures S5A,B) and chemokines (Supplementary Figures S5C,D).

**FIGURE 8:**
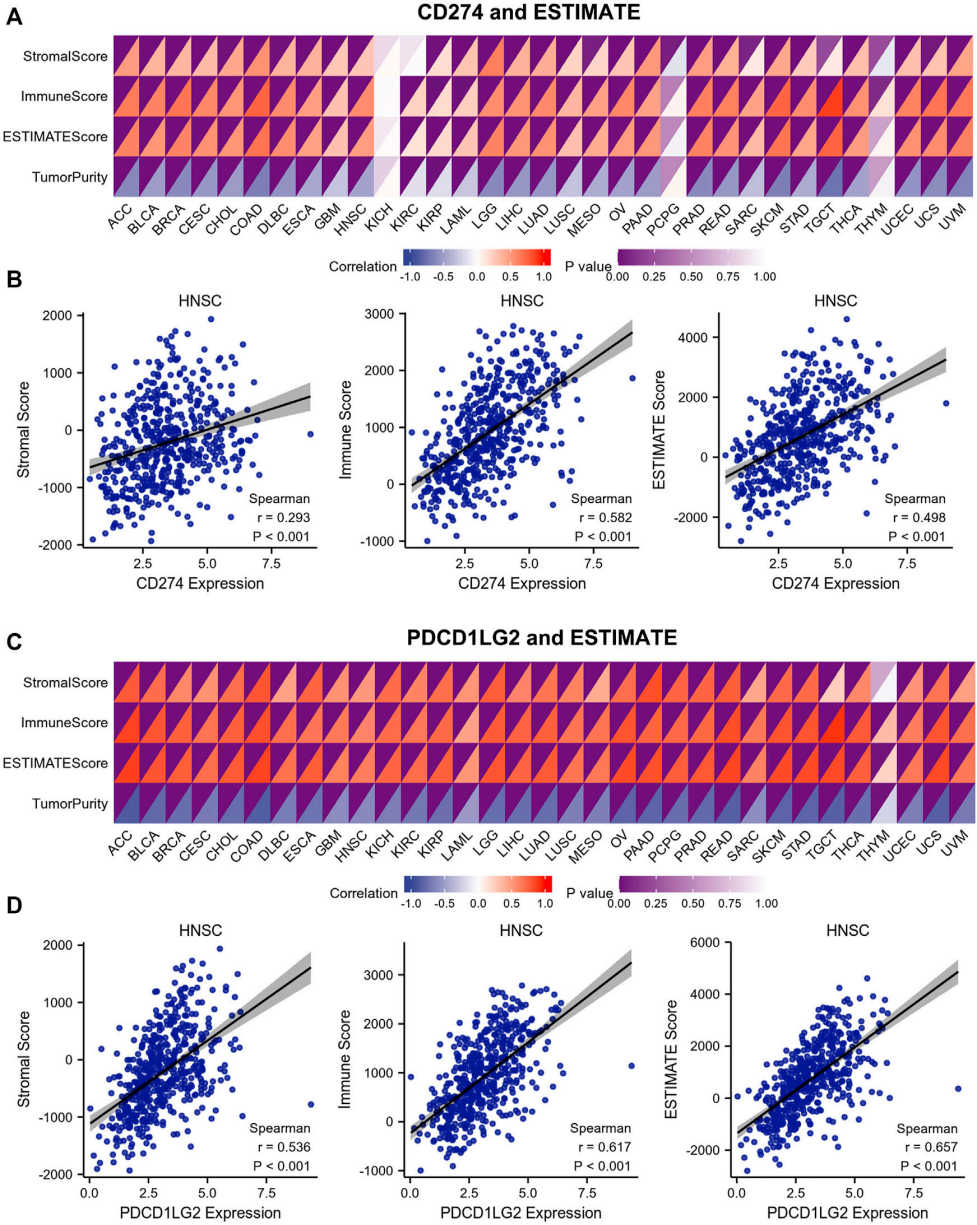
Correlations between stromal score, immune score, ESTIMATE score, and tumor purity and CD274/PDCD1LG2 levels. **(A,C)** Immune infiltration analysis of CD274 and PDCD1LG2 was performed by ESTIMATE. The color depth in the upper left corner of the correlation heatmap represents the *p* value, and the color depth in the lower right corner represents the correlation. **(B,D)** Representative HNSC results are shown at the bottom.

ESTIMATE was used to calculate the immune/stromal scores of 33 cancers. The correlation heatmap shows the association between CD274 and PDCD1LG2 expression (Figures 8A,C; Supplementary Table S3). The figures at the bottom show the typical results for HNSC (Figures 8B,D). The results indicated that CD274/PDCD1LG2 expression was positively correlated with stromal, immune, and ESTIMATE scores but negatively correlated with tumor purity.

### Correlations between CD274/PDCD1LG2 and TMB, MSI, DNMT and MMR expression

**FIGURE 9:**
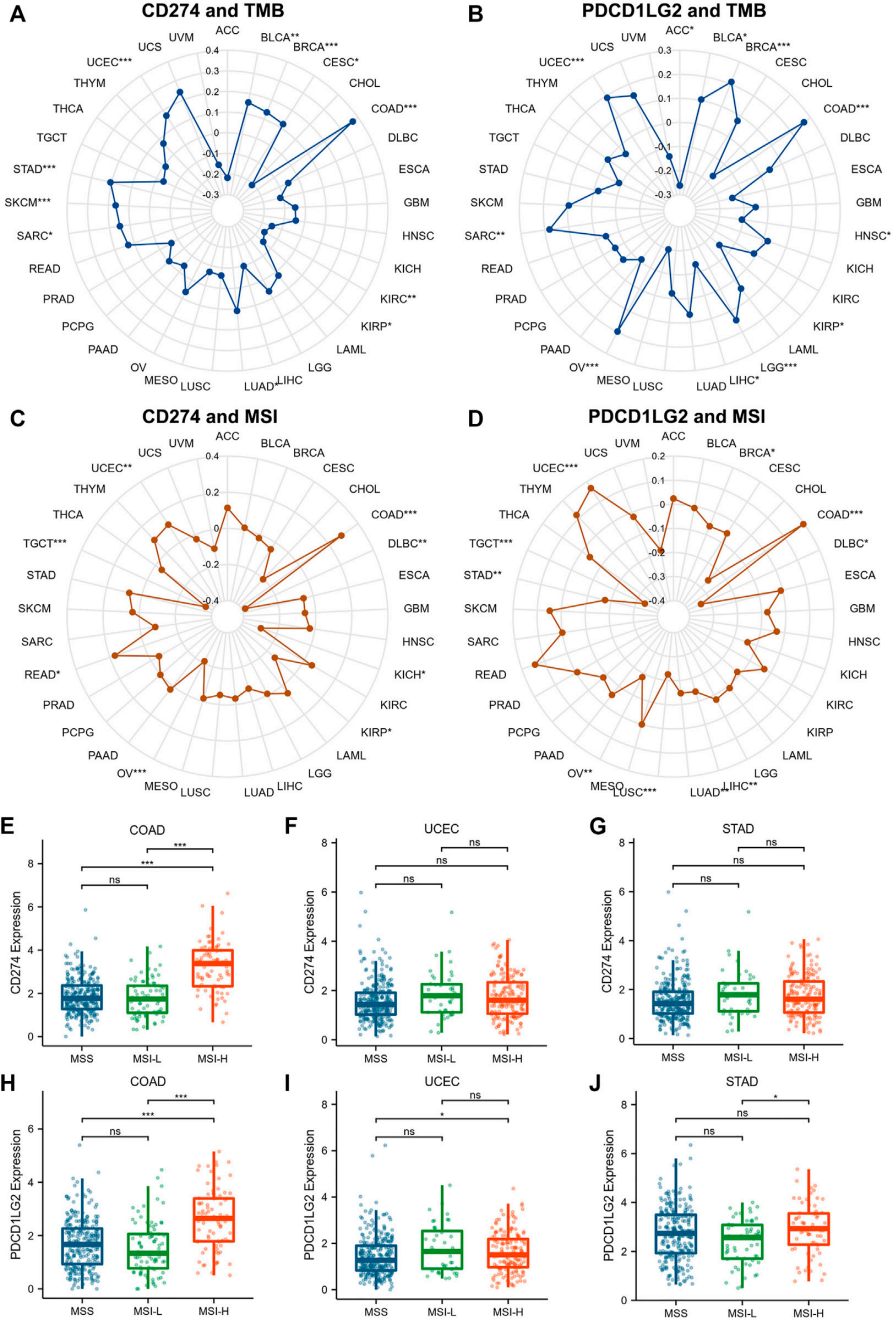
Correlations between CD274/PDCD1LG2 levels and TMB and MSI. The radar diagram indicates correlations between CD274/PDCD1LG2 levels and **(A,B)** TMB and **(C,D)** MSI, with black values representing correlation coefficients. Box plot showing the association between CD274/PDCD1LG2 levels and MSI status in **(E,H)** COAD, **(F,I)** UCEC, and **(G,J)** STAD (*p < 0.05, **p < 0.01, ***p < 0.001).

Since TMB, MSI, DNMT and MMR expression were proved associated with the efficiency of checkpoint-blocked therapy across various cancer types, the relationships between CD274/PDCD1LG2 and these markers were examined (Supplementary Table S4, S5). The radar diagrams exhibited that CD274 level was correlated with TMB in BLCA, BRCA, CESC, COAD, KIRC, KIRP, LUAD, sarcoma (SARC), SKCM, STAD, and UCEC (Figure 9A). The PDCD1LG2 level was associated with TMB in ACC, BLCA, BRCA, COAD, HNSC, KIRP, LGG, LIHC, OV, SARC, and UCEC (Figure 9B). The CD274 level was correlated with MSI in COAD, DLBC, KICH, KIRP, OV, READ, TGCT, and UCEC (Figure 9C). The PDCD1LG2 level was correlated with MSI in BRCA, COAD, DLBC, LIHC, LUAD, LUSC, OV, STAD, TGCT, and UCEC (Figure 9D). The expression levels of CD274 and PDCD1LG2 in MSI-H patients were significantly higher than those in microsatellite stable (MSS) and MSI-low (MSI-L) patients in COAD, while the correlation in UCEC and STAD was not obvious (Figures 9E–J). In addition, correlations between CD274/PDCD1LG2 and five MMR genes (MLH1, MSH2, MSH6, PMS2, and EPCAM) are displayed in Supplementary Figure S6A,B, and correlations between CD274/PDCD1LG2 expression and DNA methylation regulatory genes (DNMT1, DNMT3A, DNMT3B and DNMT3L) are displayed in Supplementary Figures S6C,D.

### Enrichment analysis

**FIGURE 10:**
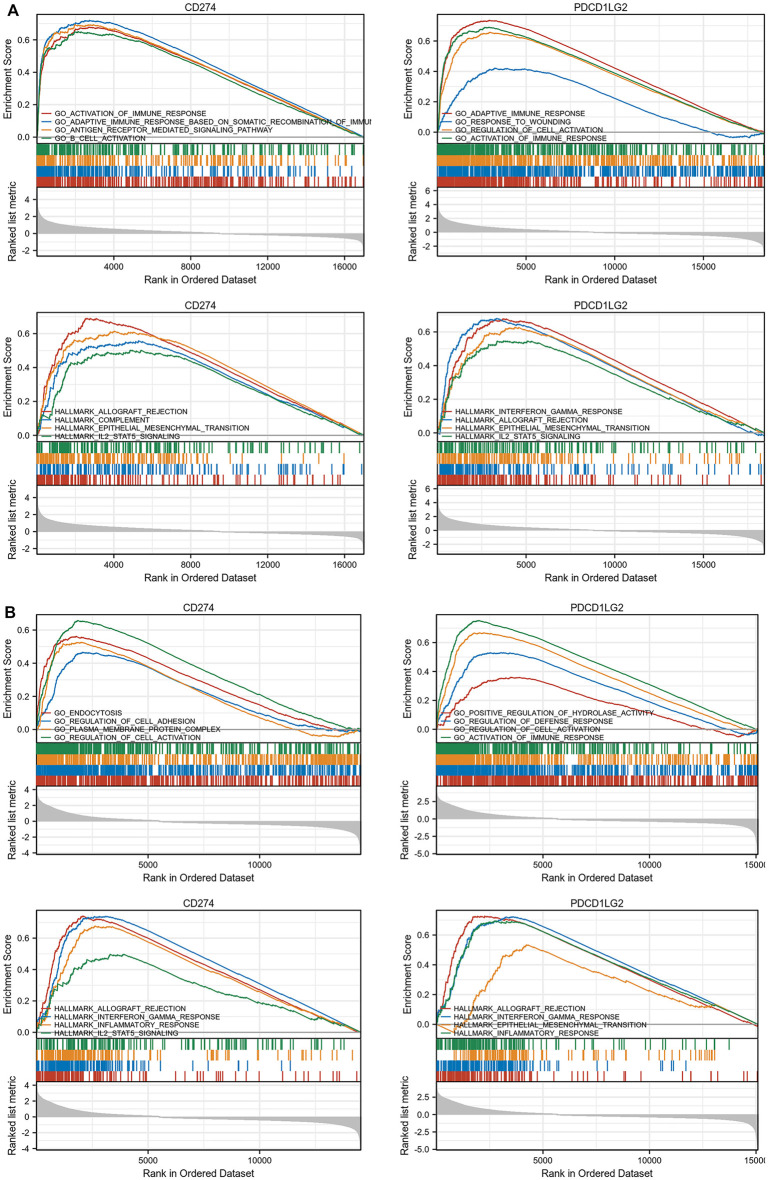
(Continued).

As CD274/PDCD1LG2 levels were found related with the clinical characteristics of LGG and SKCM, GO, KEGG analysis, and GSEA were performed for these two cancers (Figures 10A,B; Supplementary Figure S7 **)**. The results of GSEA indicated that CD274 and PDCD1LG2 were associated with several irreplaceable biological pathways, such as “Activation of Immune Reactions,” “Regulation of Cell Adhesion and Activation,” “IL2-STAT5 signaling,” and “Epithelial Mesenchymal Transition.” In addition, the results of GO and KEGG analyses indicated that CD274 and PDCD1LG2 played crucial roles in cytokine activation, cytokine and cytokine receptor interactions, and T cell activation, suggesting that CD274 and PDCD1LG2 participate in the anti-tumor immunity and cancer metastasis.

## Discussion

Recently, several clinical trials have shown that immune checkpoint inhibitors targeting the PD-1/PD-L1 axis exhibit robust antitumor efficacy in multiple advanced cancers (Finn et al., 2020; Galsky et al., 2020; Gutzmer et al., 2020; Herbst et al., 2020). However, the improvement of targeted therapies and the evaluation of efficacy still need to be emphasized (Kiyotani et al., 2021). With a better understanding of the immunotolerance mechanism, it is believed that the TPS or CPS scoring system is insufficient to predict the efficacy of anti-PD-1 therapy (Bluestone and Anderson, 2020). In the current study, we assessed the expression of CD274 and PDCD1LG2 and analyzed their associations with clinical characteristics across 33 cancer types. Interestingly, the expression levels of CD274 and PDCD1LG2 differed quite much among these cancer types, identified by TCGA databases. For instance, CD274 was upregulated in KICH and SKCM, whereas PDCD1LG2 was downregulated in KICH and SKCM. Moreover, the HPA database (https://www.proteinatlas.org/) was also used to explore their protein levels, we found that CD274 protein expression levels were higher in CESC, DLBC, HNSC, LUAD, LUSC, PAAD, SKCM, STAD, and TGCT. And PDCD1LG2 protein expression levels were higher in CESC, GBM, HNSC, LUAD, LUSC, LIHC, SKCM, OV, and TGCT. Moreover, diagnostic values of CD274 and PDCD1LG2 in various cancers were confirmed using ROC curves. Furthermore, high expression levels of CD274 and PDCD1LG2 were restrictively associated with lymph node metastasis in THCA and found to be associated with tumor stage in BLCA. Interestingly, we observed that CD274 and PDCD1LG2 expression levels were correlated with contradictory prognoses in different cancers. For instance, patients with high CD274 expression had worse prognoses in LGG, whereas those with KIRC and SKCM had better prognoses. More importantly, we found that patients with high expression of CD274 and PDCD1LG2 had better overall survival in ACC and SKCM, and patients with high expression of CD274 and PDCD1LG2 had worse overall survival in LGG, we suggested that integrate CD274 and PDCD1LG2 could better predict the prognosis in these cancers. Plenty of evidence have shown that PD-L1 (encoded by CD274) serves critical roles during tumor immune escape as well as tumor invasion and progression, and was proved to be valuable biomarker to predict worse prognosis for human cancers (Qian et al., 2018; Ren et al., 2020; Yi et al., 2021). In addition, our published data confirmed that the over expression of PD-L2 (encoded by PDCD1LG2) was negatively associated with the survival and rendered anti-EGFR resistance in HNSC patients (Qiao et al., 2021). Interestingly, in the present study, CD274 and PDCD1LG2 were found positively associated with the prognosis of several malignances, totally based on the TCGA database. Since the analysis was mainly focused on the mRNA expression, we should be concerned about the post transcriptional regulation and post translational modification of CD274 and PDCD1LG2 to discuss this complicated issue. Moreover, further proteomics analysis and clinical trials are also needed to better understand the exact prognostic value of these two biomarkers.

TME is complex and continuously evolving. In addition to endothelial cells, TME comprises innate and adaptive cells (Hinshaw and Shevde, 2019). TME is the basis of tumorigenesis and progression through interactions among cancer cells, immune cells, fibroblasts, and stromal cells (Quail and Joyce, 2013). Identification of the composition of the tumor immune microenvironment (TIME) is crucial for enhancing the efficiency of anticancer immunotherapy (Binnewies et al., 2018). Invasion of tumor-infiltrating lymphocytes (TILs) is a prerequisite for checkpoint therapy (Tang et al., 2016). Here, we evaluated the degree of immune cell infiltration across cancers using TIMER and CIBERSORT algorithms. The results demonstrated that CD274 and PDCD1LG2 expression was significantly correlated with CD8^+^ T cells, macrophages, dendritic cells, and neutrophils in most tumors, which requires further validation by experiments or single-cell sequencing. Furthermore, defective expression of MHC molecules may lead to tumor immune evasion or resistance to ICB therapy (Yamamoto et al., 2020), we found that CD274 and PDCD1LG2 were globally correlated with several immunosuppressive molecules, MHC molecules, and chemokines across cancers. To better understand the components of tumor tissue, ESTIMATE was applied, which indicated that elevated expression of CD274 and PDCD1LG2 may enrich tumor cells as well as immune cells.

Many mutations contribute to tumorigenesis and cancer progression, but the characteristics of mutations vary from each other (Iorio et al., 2016; Nusinow et al., 2020). According to published data, TMB was proven to be a crucial biomarker during immunotherapy that could predict the response to anti-PD-1 therapeutic strategies and patient prognosis (Benayed et al., 2019; Samstein et al., 2019), and patients with TMB-H generally exhibit better immunotherapy outcomes (Wang et al., 2019). We found that CD274 expression was significantly correlated with TMB in BRCA, COAD, SKCM, STAD, and UCEC, and PDCD1LG2 expression was associated with TMB in BRCA, COAD, LGG, OV, and UCEC. Moreover, MSI is reportedly irreplaceable during immune checkpoint blockade therapy, and checkpoint inhibitors were proven to be more effective in MSI-H tumors in UCEC (Arora et al., 2018). CD274 and PDCD1LG levels significantly correlated with MSI status in COAD, indicating that CD274 and PDCD1LG2 may indirectly influence immunotherapeutic effects. Additionally, MMR deficiency interferes with treatment response (Higuchi et al., 2020). Aberrant regulation of methylation may induce tumorigenesis and cancer progression (Tost, 2009; Higuchi et al., 2020). The results indicated that CD274 and PDCD1LG2 levels were highly correlated with five MMR genes and four methylation regulatory genes, the specific mechanism of which requires further research. Finally, the enrichment analysis suggested that CD274 and PDCD1LG2 were not only involved in pan-cancer immunomodulatory regulation but also contributed to tumor metastasis.

This study was subjected to some limitations. It mainly relied on public bioinformatics databases and open patient information and lacked actual clinical data and experimental validation. Furthermore, owing to a lack of protein-related data, the results of this study need to be further verified by proteomics.

In summary, our integrated analysis suggests that CD274 and PDCD1LG2 are suitable biomarkers for pan-cancer diagnostics. CD274 and PDCD1LG2 are globally related to T cell infiltration and the composition of the immunosuppressive microenvironment. We believe that this research provides a comprehensive and in-depth study of CD274 and PDCD1LG2 in genomics, which suggests that targeting PD-L1 or PD-L2 in clinical settings may be beneficial for immunotherapy.

## Data Availability

The original contributions presented in the study are included in the article/Supplementary Material, further inquiries can be directed to the corresponding authors.
