# Supplementary figures and images for: Integrative study reveals the prognostic and immunotherapeutic value of CD274 and PDCD1LG2 in pan-cancer
Source: Front Genet. 2022 Oct 6;13:990301. doi: 10.3389/fgene.2022.990301 (PMC9582533; doi:10.3389/fgene.2022.990301)

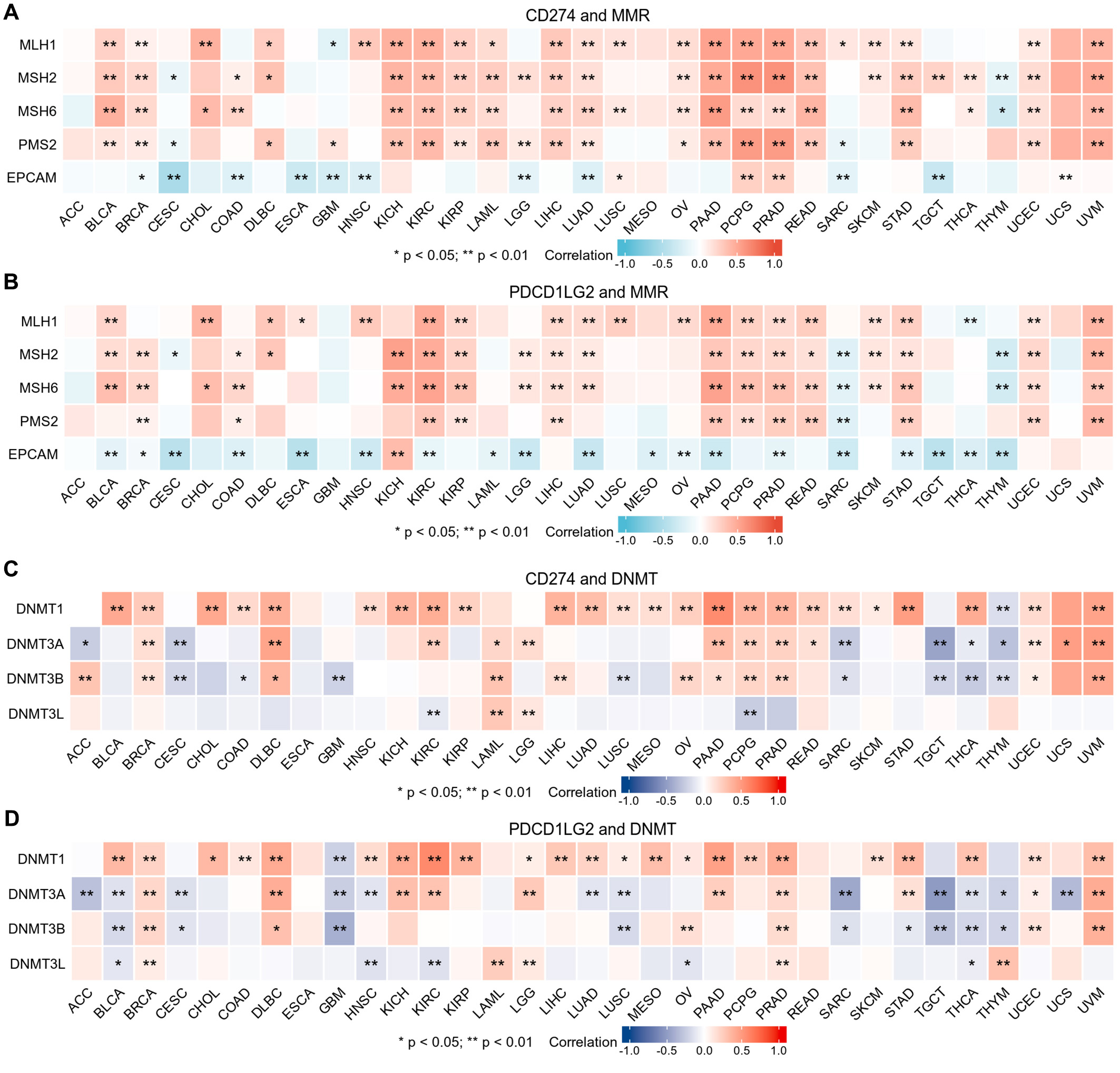

Supplement: Supplementary file 3 [file Image6.TIF]

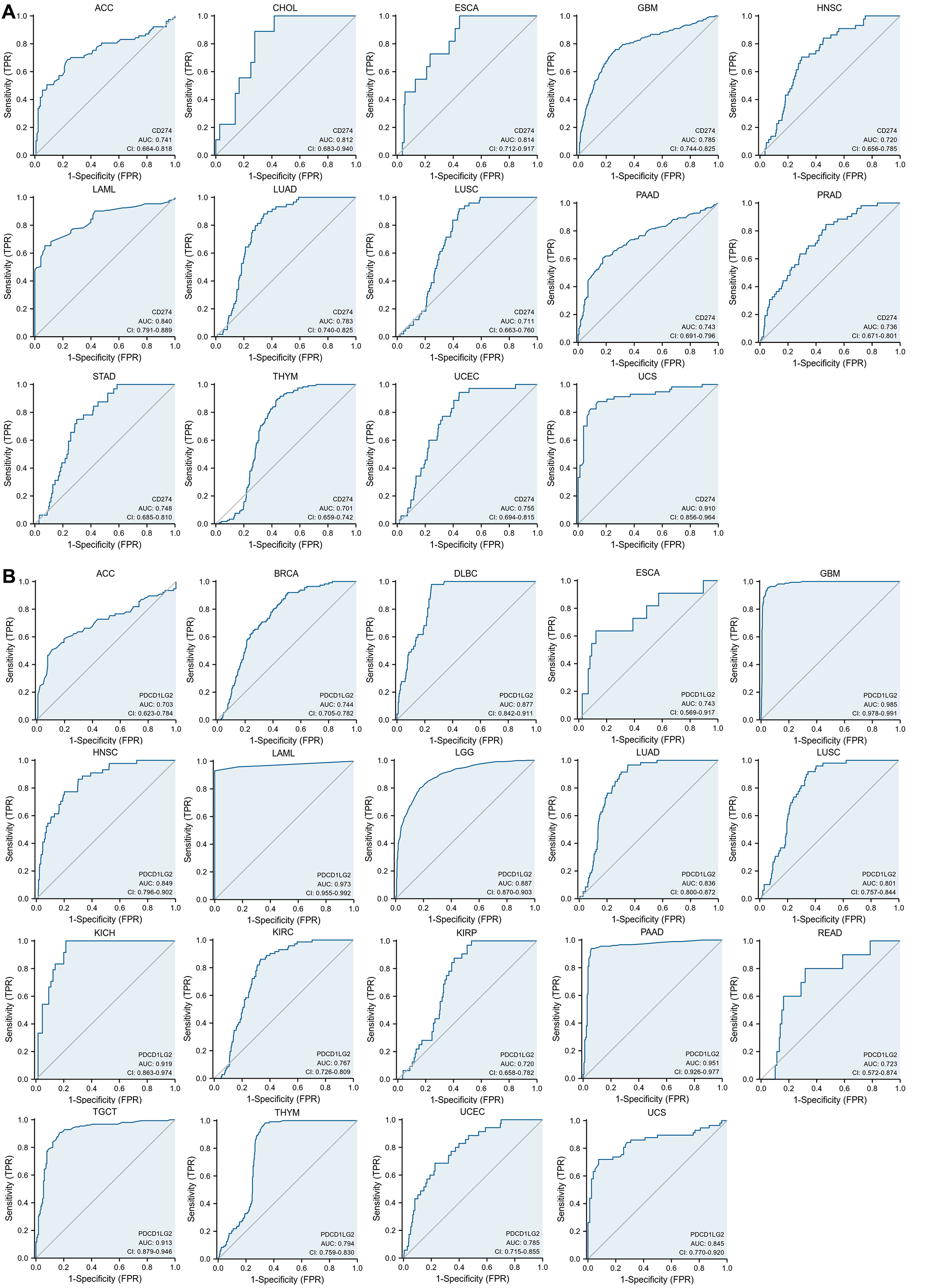

Supplement: Supplementary file 4 [file Image3.TIF]

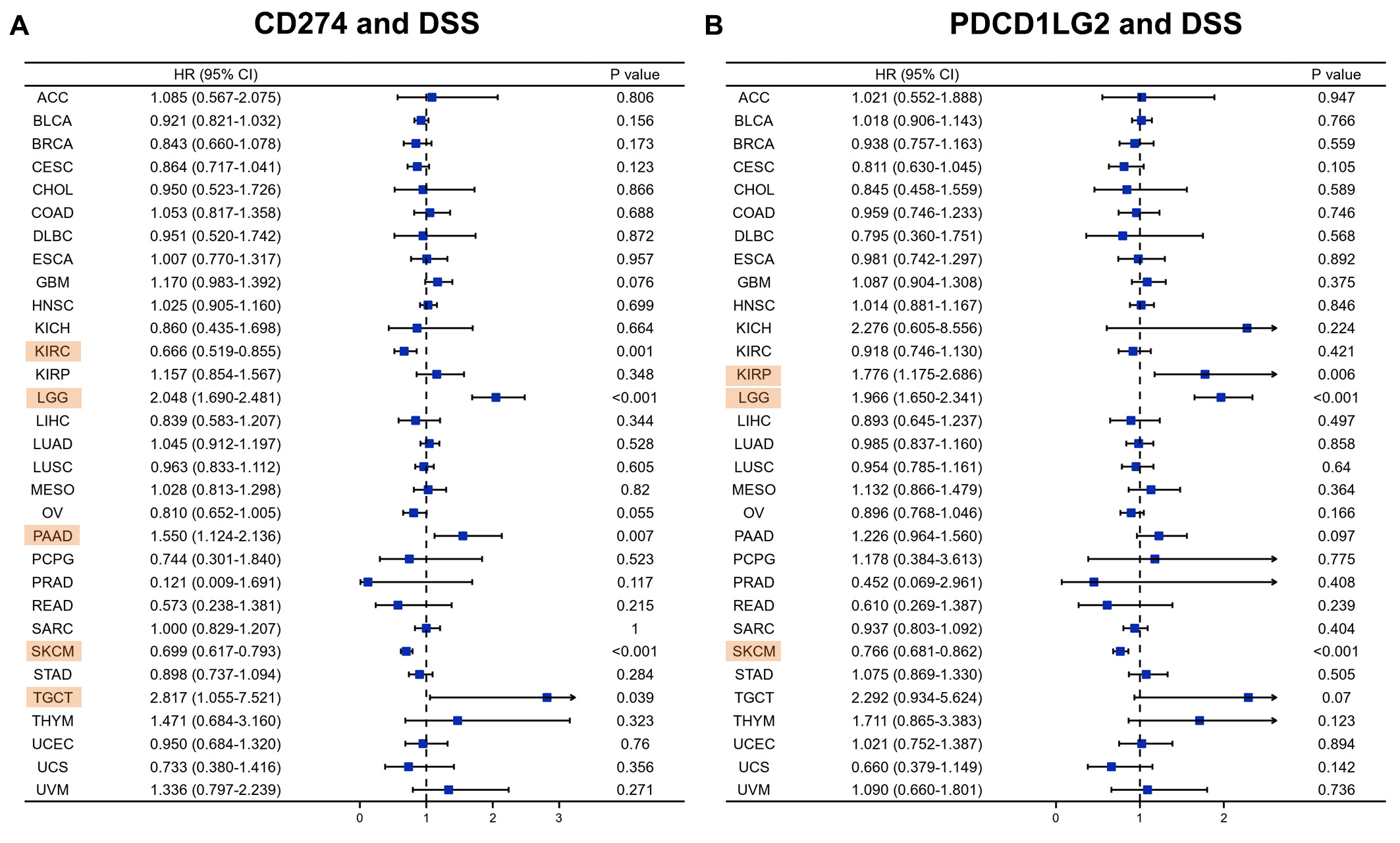

Supplement: Supplementary file 5 [file Image4.TIF]

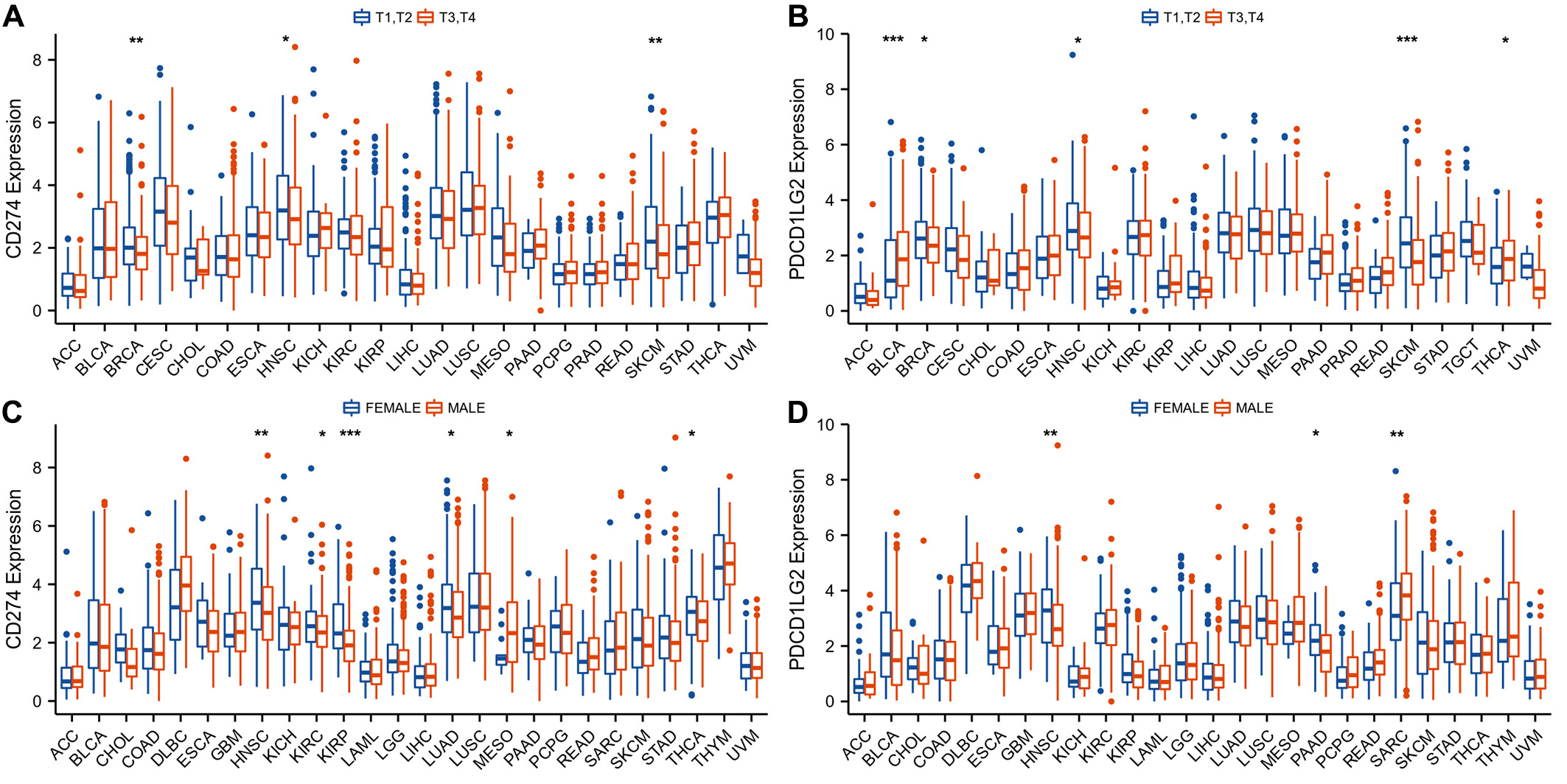

Supplement: Supplementary file 6 [file Image2.TIF]

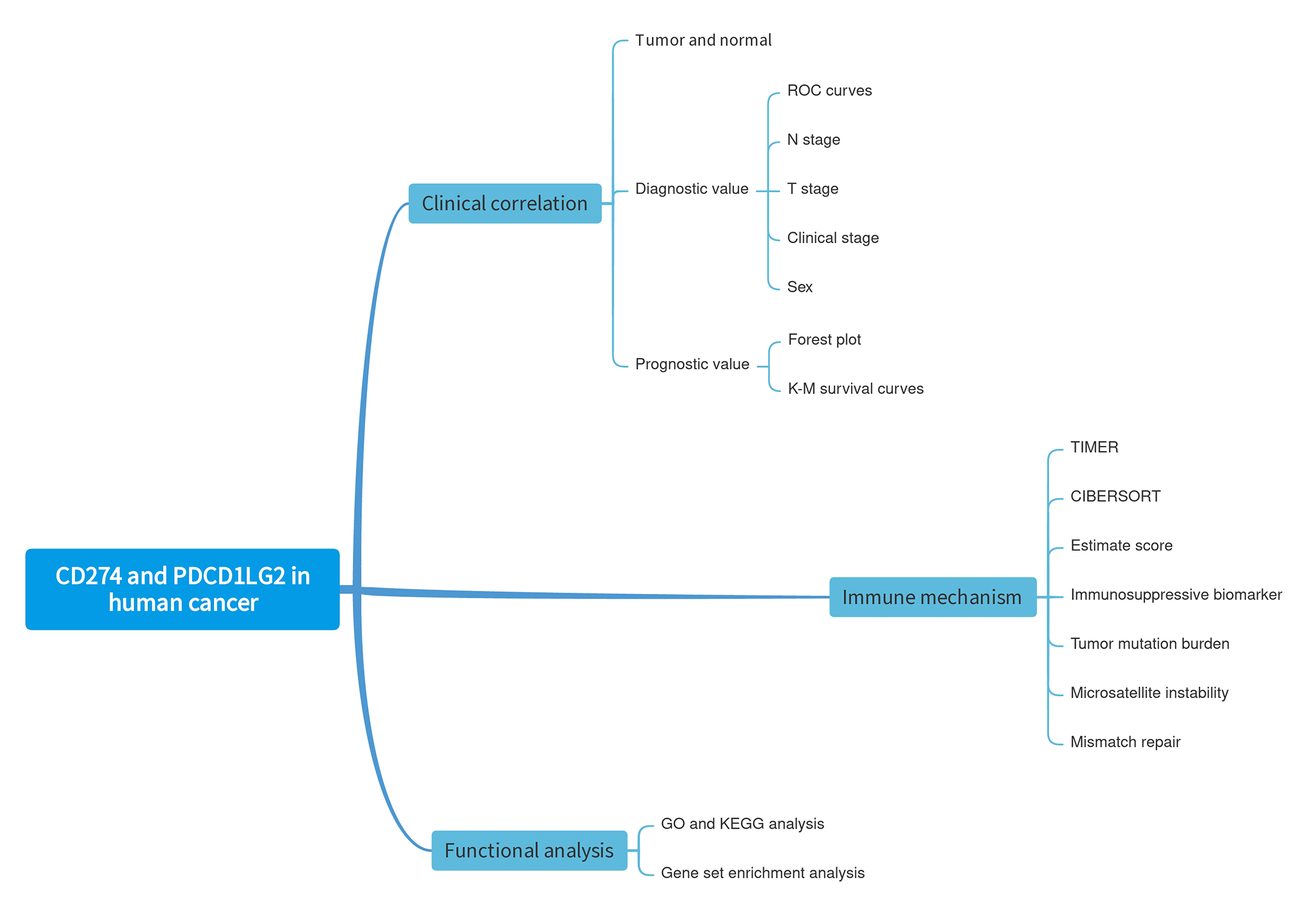

Supplement: Supplementary file 7 [file Image1.TIF]

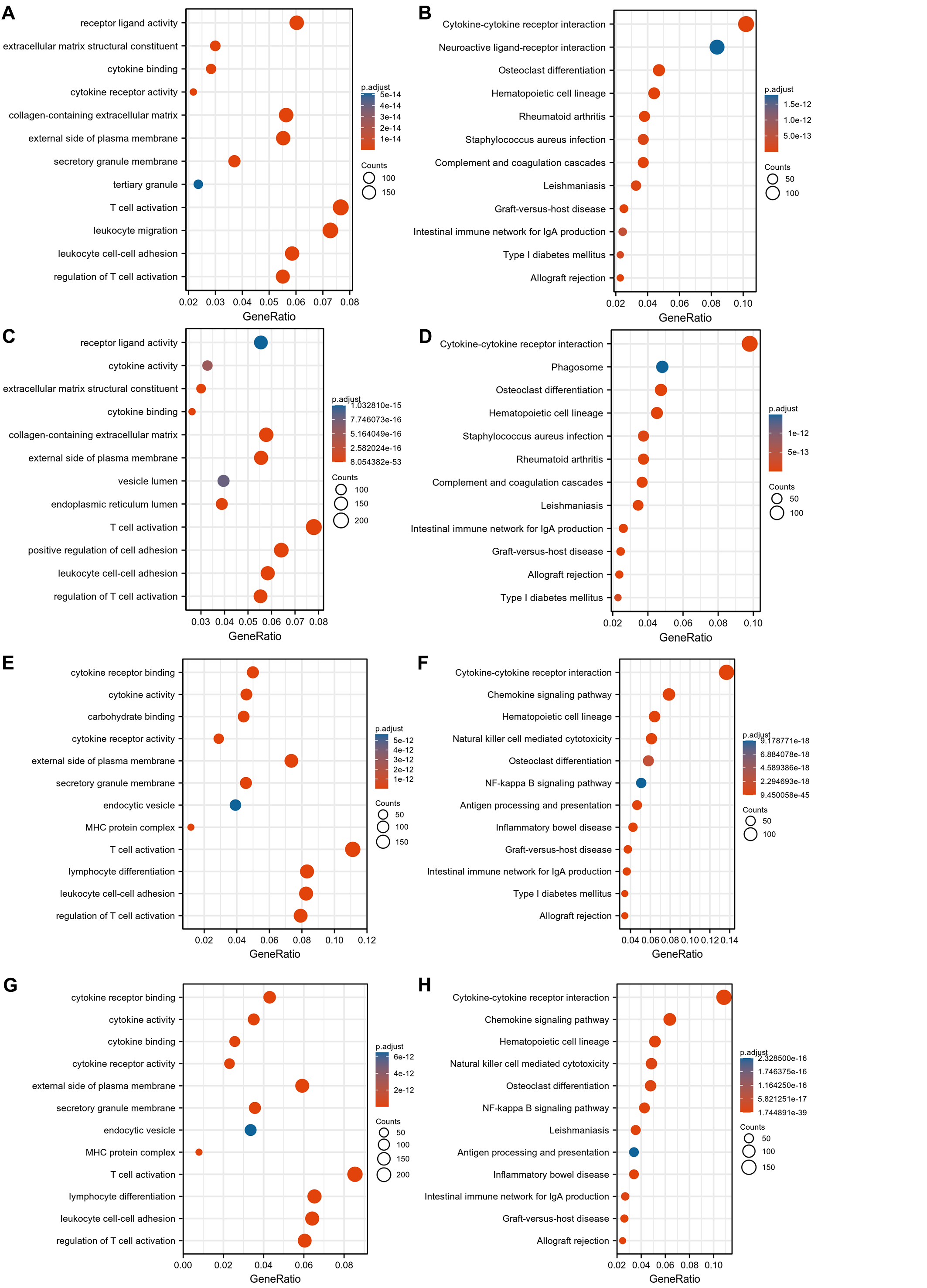

Supplement: Supplementary file 8 [file Image7.TIF]

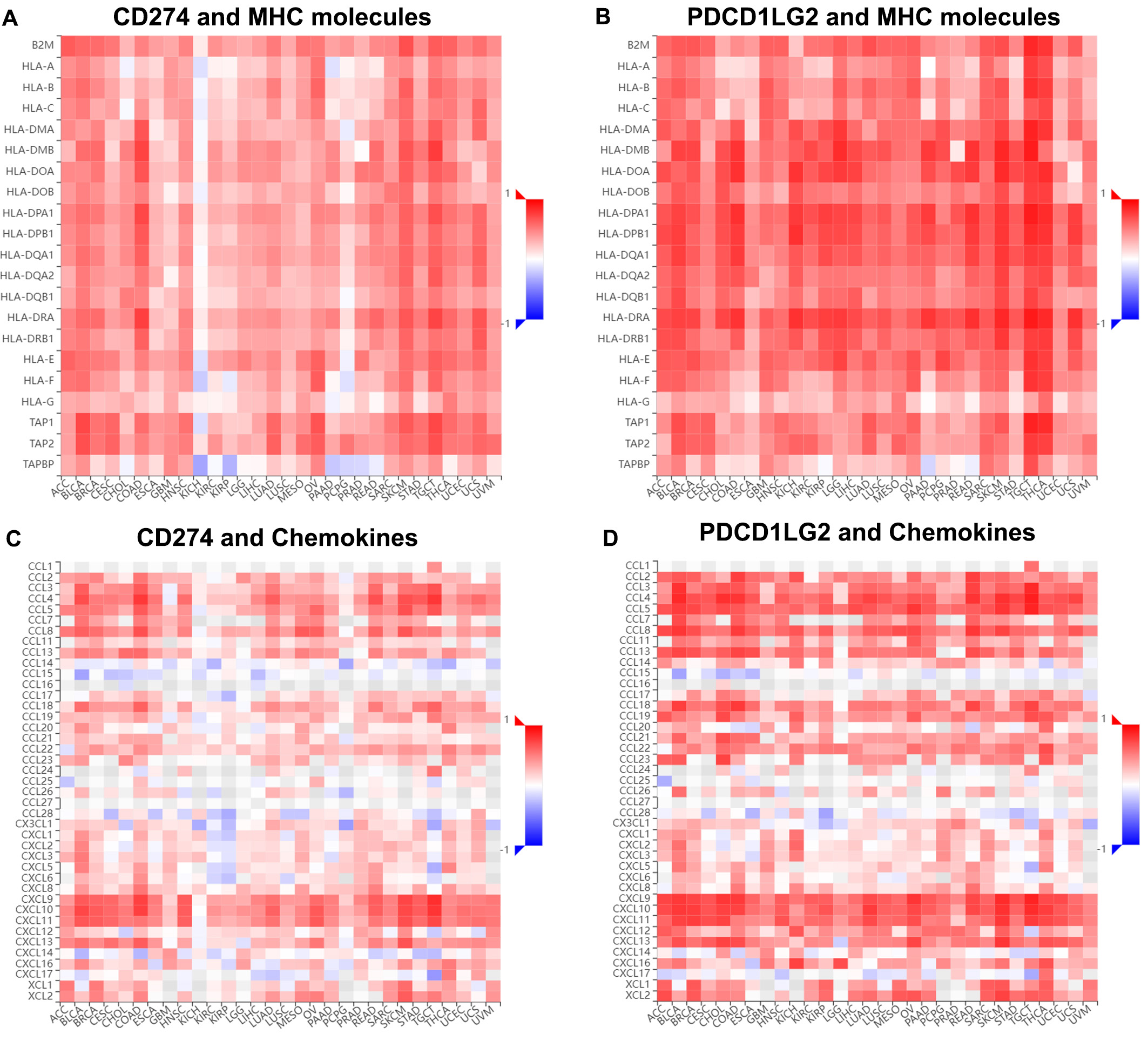

Supplement: Supplementary file 12 [file Image5.TIF]
