# Supplementary material for: Integrative study reveals the prognostic and immunotherapeutic value of CD274 and PDCD1LG2 in pan-cancer
Source: Front Genet. 2022 Oct 6;13:990301. doi: 10.3389/fgene.2022.990301 (PMC9582533; doi:10.3389/fgene.2022.990301)
Supplement: Supplementary file 14 [file DataSheet1.docx]

**Supplementary Figure Legend**

**Supplementary Figure 1**

Content and findings of this study. We compared the expression of CD274 and PDCD1LG2 in tumor and normal tissues, different N stage, T stage, clinical stage, and sex and confirmed their diagnostic value using ROC curves. Forest plots and K–M curves were used to explore their prognostic value. Multiple databases and algorithms have been used to evaluate the immune mechanisms. GO, KEGG, and GSEA were applied for functional analysis.

**Supplementary Figure 2**

Associations between CD274/PDCD1LG2 levels and clinical characteristics. **(A, B)** T stage. **(C, D)** Sex.

**Supplementary Figure 3**

Receiver operating characteristic (ROC) curves for CD274 and PDCD1LG2 expression in pan-cancer. **(A)** CD274 exhibited suitable diagnostic prediction value for adrenocortical carcinoma (ACC), cholangiocarcinoma (CHOL), esophageal carcinoma (ESCA), glioblastoma multiforme (GBM), head and neck squamous cell carcinoma (HNSC), acute myeloid leukemia (LAML), lung adenocarcinoma (LUAD), lung squamous cell carcinoma (LUSC), pancreatic adenocarcinoma (PAAD), prostate adenocarcinoma (PRAD), stomach adenocarcinoma (STAD), thyroid carcinoma (THYM), uterine corpus endometrial carcinoma (UCEC), and uterine carcinoma (UCS). **(B)** PDCD1LG2 exhibited suitable diagnostic prediction value for ACC, breast invasive carcinoma (BRCA), lymphoid neoplasm diffuse large B-cell lymphoma (DLBC), ESCA, GBM, HNSC, LAML, brain lower grade glioma (LGG), LUAD, LUSC, kidney chromophobe (KICH), kidney renal cell carcinoma (KIRC), kidney renal papillary cell carcinoma (KIRP), PAAD, rectum adenocarcinoma

(READ), TGCT, THYM, UCEC, and UCS.

**Supplementary Figure 4**

Association between CD274/PDCD1LG2 levels and disease-specific survival (DSS). Forest plot showing the association of **(A)** CD274 and **(B)** PDCD1LG2 with DSS.

**Supplementary Figure 5**

Correlations between CD274/PDCD1LG2 levels and major histocompatibility complex (MHC) molecules and chemokines. Heatmap of correlations between **(A)** CD274 and **(B)** PDCD1LG2 and MHC molecules. Heatmap of correlations between **(C)** CD274 and **(D)** PDCD1LG2 and chemokines.

**Supplementary Figure 6**

Correlations between CD274/PDCD1LG2 levels and mismatch repair (MMR) genes and DNA methylation regulatory genes. **(A, B)** Spearman correlation between CD274/PDCD1LG2 levels and MMR genes. **(C, D)** Spearman correlation between CD274/PDCD1LG2 expression and DNA methylation regulatory genes.

**Supplementary Figure 7**

Gene ontology (GO) and Kyoto Encyclopedia of Genes and Genomes (KEGG) analysis of CD274 and PDCD1LG2 in LGG and SKCM. **(A)** GO pathways of CD274 in LGG. **(B)** KEGG pathways of CD274 in LGG. **(C)** GO pathways of PDCD1LG2 in LGG. **(D)** KEGG pathways of PDCD1LG2 in LGG. **(E)** GO pathways of CD274 in SKCM. **(F)** KEGG pathways of CD274 in SKCM. **(G)** GO pathways of PDCD1LG2 in SKCM. **(H)** KEGG pathways of PDCD1LG2 in SKCM.
